# Maternal function and clinical implications: case report

**DOI:** 10.1192/j.eurpsy.2022.2222

**Published:** 2022-09-01

**Authors:** I. Cuevas Iñiguez, M.D.C. Molina Liétor

**Affiliations:** 1 Hospital Principe de Asturias, Psiquiatría, Alcala de Henares, Spain; 2 Hospital Universitario Príncipe de Asturias, Psiquiatría, Alcalá de Henares, Spain

**Keywords:** motherhood

## Abstract

**Introduction:**

Multiple authors have criticized the lack of attention that classical theoretical models have paid to motherhood as a milestone of great influence on the psychic structure of women. However other models have developed theories that take into account factors such as: motherhood implies “dying as a daughter” or the oscillations between the “desire of the mother” and the “desire of the woman”.

**Objectives:**

This case report aims to describe a case of severe difficulties achieving maternal function.

**Methods:**

Case report and literature review.

**Results:**

A 27 years old woman, born in Ethiopia. The patient reported history of childhood trauma (intrafamiliar sexual abuse, child neglect). Depressed mood and pasive autolytic ideation since childhood. The patient was adopted when she was 11 years old and moved to Spain. The pacient had difficulties with bonding with her adoptive family. At the age of 24, she got pregnant “to have my own family and not being alone.” During pregnancy, she begins to present poorly structured paranoid ideation. After birth, the patient began to present autolytic ideation, dissociative symptoms and suicide attempts.

**Conclusions:**

For the patient, her desire to be a mother, marked from the beginning by the phantom of appropriation, later led to rejecting it. Various factors could affect: her motherhood resignified the relationship with her family of origin, as well as having imagined that her daughter would complete her lack: the birth could have underlined her traumatic history, marking the bond with her daughter by indifference and the lack of libidinal investiture.

**Disclosure:**

No significant relationships.

